# 

**Published:** 1813-08

**Authors:** 


### MONTHLY CATALOGUE OF MEDICAL BOOKS.

A PRACTICAL Treatise on the Remittent Fever of Infants; with Remarks on Hydrocephalus Internus, or Water in the Brain; and several other Diseases: and Cases and Observations designed to illustrate the influence exerted by a certain disordered State of the Chylopoietic Viscera upon local and constitutional Diseases; and to prove the Utility and Necessity of removing it, in order to facilitate and establish their Cure. In Two Parts. By James Millman Coley, Member of the Royal College of Surgeons, &c. &c. 8vo.—Underwood.

The Modern Practice of Physic; exhibiting the Character, Causes, Symptoms, Prognostic, Morbid Appearances, and improved Method, of treating the Diseases of all Climates. By Robert Thomas, M.D. Fourth edition, revised and considerably enlarged. 8vo.

Medical Histories and Reflections, Vol. 4. By John Ferriar, M.D. 8vo.—Cadell and Co.

A short Account of Experiments and Instruments depending on the Relations of Air to Heat and Moisture. By John Leslie, F.R.S.E. 8vo.—Longman and Co.

A Letter addressed to Mr. U. T. Hausmann, by J. A. de Luc, Esq. giving an Account of the Origin of Hygrometry, of the Invention of the Hygrometer, and of its practical Use; to which are added, Tables expressing the quantities of Water contained in a given space of Air for each degree of that Hygrometer and the Thermometer, constructed by U. T. Hausmann. 4to.—Hatchard.

---

### *NOTICES TO CORRESPONDENTS.*

*We have received several letters upon the subject of Medical Reform; from the general feeling expressed in them, it appears to be the wish of some respectable correspondents, that the discussion should still be continued, presuming that the bill will again be brought before Parliament. We shall therefore take an early occasion for renewing the subject.*

*Mr. Pulley and Mr. Gibbon again call upon Dr. Yeats to publish the case of Ann Foulkes. From their statement, it would seem as if the doctor had been imposed upon.—Mr. Pulley writes, "I still feel myself under the necessity of publicly calling on Dr. Yeats for his case of vomiting of urine. His pledge to publish it cannot be considered in the light of a* private *intimation, nor was it* loosely *given; it was positive and distinct; and his determination to send it to the world arose from the difference of opinion entertained by other medical men."—Mr. Gibbon considered the case to be a gross instance of imposture, and his opinion appears to be sanctioned by the most eminent practitioners in the neighbourhood of Bedford, where the peculiar circumstances of the case have excited an extraordinary degree of interest.*

